# Neural Conduction Along Postretinal Visual Pathways in Glaucoma

**DOI:** 10.3389/fnagi.2021.697425

**Published:** 2021-08-02

**Authors:** Vincenzo Parisi, Lucia Ziccardi, Lucia Tanga, Gloria Roberti, Lucilla Barbano, Carmela Carnevale, Gianluca Manni, Francesco Oddone

**Affiliations:** ^1^IRCCS—Fondazione Bietti, Rome, Italy; ^2^Department of Clinical Sciences and Translational Medicine, University of Rome Tor Vergata, Rome, Italy

**Keywords:** glaucoma, visual pathways, PERG, VEP, OCT imaging

## Abstract

**Purpose**: This study was conducted in order to evaluate retinal ganglion cell (RCG) function and the neural conduction along the postretinal large and small axons and its correlation with retinal nerve fiber layer thickness (RNFL-T) in open-angle glaucoma (OAG) eyes.

**Methods**: Thirty-seven OAG patients (mean age: 51.68 ± 9.83 years) with 24–2 Humphrey mean deviation (MD) between −2.5 and −20 dB and IOP <21 mmHg on pharmacological treatment (OAG group) and 20 age-matched controls (control group) were enrolled. In both groups, simultaneous pattern electroretinograms (PERG) and visual evoked potentials (VEP), in response to checks stimulating macular or extramacular areas (the check edge subtended 15′ and 60′ of visual arc, respectively), and RNFL-T (measured in superior, inferior, nasal, and temporal quadrants) were assessed.

**Results**: In the OAG group, a significant (ANOVA, *p* < 0.01) reduction of 60′ and 15′ PERG P50-N95 and VEP N75-P100 amplitudes and of RNFL-T [overall (average of all quadrants) or temporal] with respect to controls was found; the values of 60′ and 15′ PERG P50 and VEP P100 implicit times and of retinocortical time (RCT; difference between VEP P100 and PERG P50 implicit times) were significantly (*p* < 0.01) increased with respect to control ones. The observed increased RCTs were significantly linearly correlated (Pearson’s test, *p* < 0.01) with the reduced PERG amplitude and MD values, whereas no significant linear correlation (*p* < 0.01) with RNFL-T (overall or temporal) values was detected.

**Conclusions**: In OAG, there is an impaired postretinal neural conduction along both large and small axons (increased 60′ and 15′ RCTs) that is related to RGC dysfunction, but independent from the RNFL morphology. This implies that, in OAG, the impairment of postretinal neural structures can be electrophysiologically identified and may contribute to the visual field defects, as suggested by the linear correlation between the increase of RCT and MD reduction.

## Introduction

Open-angle glaucoma (OAG) is actually considered a multifactorial optic neuropathy characterized by optic nerve cupping and typical visual field defects (Quigley, 2011). Several histological studies performed in experimental models and in humans suggested that, in glaucoma, there is a selective and progressive damage of retinal ganglion cells (RGC) and their fibers (retinal nerve fiber layer, RNFL; Quigley et al., 1982, 1987, 1988, 1995). More recently, it was suggested that glaucomatous disease induces damage not exclusive at the level of RGC, but also involvement of the postretinal structures, being a neurodegenerative process that extends to the lateral geniculate nucleus (LGN; Chaturvedi et al., 1993; Weber et al., 2000).

The gold standard to evaluate and follow-up visual dysfunction in OAG is the threshold static standard perimetry [i.e., Humphrey Field Analyzer (HFA) SITA Standard 24–2 strategy] (Scuderi et al., 2008). However, since HFA perimetry is based on psychophysical responses proved by visual cortex, it does not reveal which structures of the visual pathways selectively contribute to glaucomatous defects (Kothari et al., 2013).

Alternatively, electrophysiological methods allow to objectively evaluate the function of different visual pathway structures. In particular, studies in animals (Maffei and Fiorentini, 1981) and humans (Parisi, 2003) suggest that a functional integrity of RGC and their fibers is required to provide normal electroretinographic responses recorded by using patterned stimuli (pattern ERG, PERG). The function of the whole visual pathway can be assessed by recording cortical potentials evoked by patterned stimuli (visual evoked potentials—VEPs), and by using appropriate methods (check edge of 60′ and 15′ of visual arc), it is possible to evaluate selectively the neural conduction along the large and the small axons forming the visual pathways (Parisi et al., 2010, 2019a,b; Ziccardi et al., 2013; Odom et al., 2016; Barbano et al., 2021).

Actually, it is known that in OAG patients, PERG and VEP obtained in response to particular visual stimuli (80% contrast, 15′ checks reversed at the rate of 2 reversals per second) may have a specificity of 100% to detect both RGC and visual pathway dysfunction (Parisi et al., 2006); in addition, reduced PERG amplitudes were significantly correlated with the reduction of RNFL thickness (RNFL-T; Parisi et al., 2001, 2006; Ventura et al., 2006; Falsini et al., 2008; Jeon et al., 2019; Jung et al., 2020; Barbano et al., 2021), whereas the delayed VEP responses were (Moschos et al., 2012) or not (Parisi et al., 2001) correlated with the RNFL-T reduction.

At the present, single electrophysiological methods do not allow to evaluate selectively the function of specific postretinal structures. However, by using simultaneous recordings of PERG and VEP, it is possible to obtain an “electrophysiological index” of postretinal neural conduction (derived from the difference between VEP P100 and the PERG P50 implicit times), known as “retinocortical time” (RCT; Celesia and Kaufmann, 1985; Celesia et al., 1986).

In OAG patients, an increase of RCT was detected, and it was significantly linearly correlated with the RGC dysfunction (reduced PERG amplitudes; Parisi, 1997; Parisi et al., 2001). Nevertheless, in these studies, the RCT was assessed exclusively by using visual stimuli for evaluating the neural conduction along the small axons (i.e., 15′ checks). Therefore, actually, there is a lack of information about the postretinal neural conduction along the large axons and whether this is related to RGC function.

In addition, contrary to reported evidence showing the correlation between PERG, VEP, and RNFL-T (Parisi et al., 2001; Ventura et al., 2006; Falsini et al., 2008; Moschos et al., 2012; Jeon et al., 2019; Jung et al., 2020), the relationship between the postretinal neural conduction along large and small axons (assessed by RCT in response to 60′ and 15′ checks) and the morphology of RNFL was never investigated. Thus, in OAG, it is actually also unknown whether the postretinal impairment could be dependent or not from the morphological condition of RGC fibers.

Therefore, our study aimed to evaluate the neural conduction along the postretinal large and small axons (assessed by RCT in response to 60′ and 15′ checks) and whether it could be related to RCG function (assessed by PERG recordings) and/or could be dependent or not from the RNFL morphological condition in OAG eyes.

## Materials and Methods

### Participants

All research procedures described in this work adhered to the tenets of the Declaration of Helsinki. The study protocol was approved by the local IRB (Comitato Scientifico IRCCS Fondazione Bietti, Rome, Italy), and upon recruitment, informed consent after full explanation of the procedure was obtained from each subject enrolled in the study.

Thirty-seven consecutive patients affected by OAG were recruited and selected from a larger population of 274 patients based on the following inclusion criteria:

(1) age between 30 and 75 years.
(2) diagnosis of OAG with a repeatable HFA 24–2 SITA Standard visual field defect compatible with glaucoma and a mean deviation (MD) between −2 and −20 dB.
(3) typical glaucomatous optic nerve head damage.
(4) best corrected visual acuity ≥8/10 Snellen, and
(5) intraocular pressure (IOP) values less than 18 mmHg under topical hypotensive treatment (monotherapy as well as combined therapy) during, at least, 8 months preceding the electrophysiological and morphological evaluation. It is known that the PERG responses could be modified by the reduction of the IOP with beta-blocker treatment (Papst et al., 1984; Nesher et al., 1990; Arden and O’Sullivan, 1992; Falsini et al., 1992; Colotto et al., 1995; Parisi, 2001; Ventura and Porciatti, 2005).

The exclusion criteria were as follows:

(1) ocular surgery, including cataract surgery, in the last 3 months.
(2) cataract or macular diseases.
(3) argon laser trabeculoplasty in the last 6 months.
(4) secondary ocular hypertension, including steroid-induced ocular hypertension.
(5) ocular or systemic diseases that could affect the outcome of the study.
(6) changes of systemic treatments that could affect IOP values.
(7) treatment with lutein, zeaxanthin, citicoline, docosahexaenoic acid, ubiquinone, or coenzyme Q10 in the last 3 months.
(8) pregancy, breastfeeding.
(9) diabetes.
(10) systemic lupus erythematosus, rheumatoid arthritis, and connectivitis; and
(11) use of anticoagulants and lithium.

### Electrophysiological (PERG and VEP) Assessment

We performed simultaneous PERG and VEP recordings by using Retimax Advanced Plus apparatus (CSO, Firenze, Italy) and according to previously published methods (Parisi, 1997, 2001; Parisi et al., 2001, 2006, 2010, 2019a,b; Ziccardi et al., 2013; Barbano et al., 2021).

Briefly, the visual stimulation was monocular after occlusion of the other eye, and the visual stimuli were checkerboard patterns (contrast, 80%; mean luminance, 110 cd/m^2^) generated on a TV monitor and reversed in contrast at the rate of two reversals per second. At the viewing distance of 114 cm, the check edges subtended 60 min (60′) and 15 min (15′) of the visual angle. We used two different checkerboard patterns, as suggested by the VEP ISCEV standards (Odom et al., 2016) to obtain a prevalent activation of larger (60′ checks) or smaller (15′ checks) axons (Parisi et al., 2010, 2019a,b; Ziccardi et al., 2013; Barbano et al., 2021). The monitor screen subtended 23°. A small fixation target, subtending a visual angle of approximately 0.5° (estimated after taking into account spectacle-corrected individual refractive errors), was placed at the center of the pattern stimulus. For every PERG and VEP acquisition, each patient positively reported that he/she could clearly perceive the fixation target.

The PERG bioelectrical signal was recorded by a small Ag/AgCl skin electrode placed over the lower eyelid. PERGs were bipolarly derived between the stimulated (active electrode) and the patched (reference electrode) eye. A discussion on PERG using skin electrodes and its relationship to the responses obtained by corneal electrodes can be found elsewhere (Fiorentini et al., 1981; Hawlina and Konec, 1992; Porciatti and Falsini, 1993).

In the PERG response, we considered peaks having the following implicit times (IT): 50 and 95 ms (P50, N95); the peak-to-peak amplitude between P50 and N95 was measured (PERG A). In the VEP response, we considered and measured peaks having the following implicit times: 75 and 100 ms (N75 and P100), and the peak-to-peak amplitude between N75 and P100 was measured (VEP A). The RCT for each simultaneous recording of PERG and VEP was calculated as the difference between VEP P100 and the PERG P50 ITs (Celesia and Kaufmann, 1985; Celesia et al., 1986).

During a recording session, simultaneous PERG and VEP were recorded at least twice (between two and six times), and the resulting waveforms were superimposed to check the repeatability of the results. On the basis of our previous studies (Parisi, 1997; Parisi et al., 2001, 2006), we know that intraindividual variability (evaluated by test–retest) is approximately ± 2 ms for VEP P100 IT and approximately ± 0.18 V was required (albeit never more than six in the cohort of patients). For statistical analyses (see below), we considered PERG and VEP values measured in the recording with the lowest PERG A.

In each patient, the signal-to-noise ratio (SNR) of PERG and VEP responses was assessed by using our previously published methods (Parisi, 1997; Parisi et al., 2001, 2006, 2010, 2019a,b; Ziccardi et al., 2013; Barbano et al., 2021). We accepted VEP and PERG signals with a SNR >2 for all subjects.

### Retinal Nerve Fiber Layer Thickness Assessment

RNFL-T was assessed by using a spectral-domain optical coherence tomography (Sd-OCT) device [Heidelberg Spectralis (version 1.10.4.0), Heidelberg Engineering, Heidelberg, Germany] according to the APOSTEL recommendations (Cruz-Herranz et al., 2016). Circular scan centered on the optic nerve head (ONH) was acquired using the peripapillary RNFL protocol. The characteristics of Sd-OCT evaluation are reported extensively in our previous works (Ziccardi et al., 2013, 2015). In the Sd-OCT results, we considered the averaged values of RNFL-T from the following quadrants: superior, inferior, nasal, and temporal (RNFL-TT); the overall data obtained as the global average from all quadrants were identified as RNFL overall (RNFL-OT).

### Statistical Analysis

For PERG, VEP, and RNFL parameters, 95% confidence limits were obtained from control data by calculating mean values +2 standard deviations (SD) for PERG, VEP IT, and RCT and mean values −2 SD for PERG A, VEP A, and RNFL-T (see Table 1).

**Table 1:** Mean values of 60′ and 15′ pattern electroretinogram (PERG) P50 implicit time (IT) and P50-N95 amplitude (A), visual evoked potentials (VEP) P100 IT and N75-P100 A detected in controls and in patients with open-angle glaucoma (OAG).

	Controls (*N* = 20)		OAG (*N* = 37)		ANOVA: OAG vs. controls		Ab	%
	Mean	1 SD	Mean	1 SD	*F*(1, 56)	*p*
60′ PERG IT (ms)	54.23	3.22	62.03	2.95	85.13	<0.0001	32	86.4
60′ PERG A (μV)	2.02	0.16	0.90	0.19	501.50	<0.0001	37	100
60′ VEP IT (ms)	101.72	2.17	125.00	4.94	399.77	<0.0001	37	100
60′ VEP A (μV)	10.03	1.76	5.38	1.97	77.75	<0.0001	35	94.5
60′ RCT (ms)	47.49	2.88	63.03	4.80	174.70	<0.0001	37	100
15′ PERG IT (ms)	54.56	3.62	62.89	2.79	93.62	<0.0001	37	100
15′ PERG A (μV)	2.23	0.19	0.78	0.17	869.64	<0.0001	37	100
15′ VEP IT (ms)	103.88	2.94	127.46	4.27	483.80	<0.0001	37	100
15′ VEP A (μV)	9.73	1.64	4.59	2.13	87.93	<0.0001	37	100
15′ RCT (ms)	49.32	2.07	64.57	3.68	291.87	<0.0001	37	100

*Abbreviations: *N*, number of eyes; SD, standard deviation; ANOVA, one-way analysis of variance; Ab, abnormal with respect to 95% normal confidence limits (mean values +2 SD for VEP ITs, PERG ITs, and RCTs and mean values −2 SD for PERG and VEP As); 60′ and 15′, visual stimuli with checks subtending 60 and 15 min of visual arc, respectively; RCT, difference VEP 100 minus PERG P50 implicit times; ms, milliseconds; μV, microvolt*.

Differences of PERG, VEP, RCT, and RNFL-T values between OAG and control groups were evaluated by one-way analysis of variance (ANOVA). Pearson’s test was applied to compare electrofunctional (PERG IT and A, VEP IT and A, RCT), morphological (RNFL-T), and MD data.

In all analyses, we considered as statistically significant a *p*-value lower than 0.01. Minitab 17 (version 1) software was used for statistics.

## Results

Based on the abovementioned inclusion/exclusion criteria, 37 consecutive selected patients were enrolled in the study. There were 12 females and 25 males, with ages ranging between 34 and 70 years (mean age 51.68 ± 9.83 years).

When the same patient showed an adherence to inclusion/exclusion criteria in both eyes, the eye with the better MD was considered, and therefore, the statistical analysis (see above, “Statistical Analysis” section) was performed based on 37 eyes from 37 OAG patients.

The MD ranged from −2.58 to −19.62 dB (mean −9.84 ± 4.28 dB), and based on the Hodapp–Parrish–Anderson criteria (Hodapp et al., 1993), our cohort of OAG patients was constituted by 6 patients with early defect, 20 patients with moderate defect, and 11 patients with severe defect.

**Figure 1:**
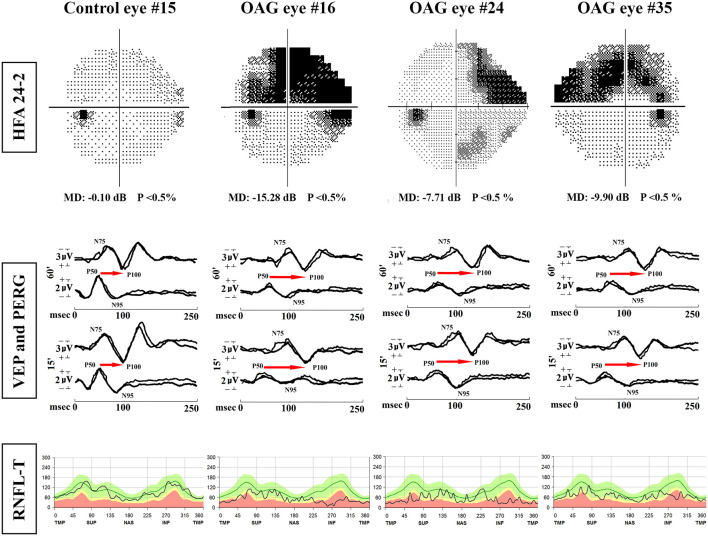
Examples of Humphrey Field Analyzer (HFA) 24-2, simultaneous pattern electroretinogram (PERG) and visual evoked potentials (VEP) recordings and retinal nerve fiber layer thickness (RNFL-T) analysis observed in one representative control eye (#15) and three representative OAG eyes (#16, #24, #35). MD, mean deviation; 60′ and 15′, check edges subtending 60 min (60′) and 15 min (15′) of the visual angle for PERG and VEP visual stimuli; N75 and P100 refer to the first negative and the first positive peak of VEP recordings (the implicit time of P100 and the peak-to-peak N75-P100 amplitude were considered); P50 and N95 refer to the first positive and the second negative peak of PERG recordings (the implicit time of P50 and the peak-to-peak P50-N95 amplitude were considered); the retinocortical time (RCT), indicated by the red arrow, is the difference between VEP P100 and PERG P50 implicit times. msec, milliseconds; μV, microvolt; T, temporal; S, superior; N, nasal; I = inferior refer to the RNFL sectorial thickness expressed in microns. In OAG eyes, with respect to the control eye, it is possible to observe reduced P50-N95 PERG and N75-P100 VEP amplitudes, delayed P50 PERG and P100 VEP implicit times, increased RCT, and reduced RNFL-T.

Representative examples of HFA 24–2, PERG and VEP recordings, and RNFL-T observed in one control eye (#7) and on three OAG eyes (#16, #24, #35) are shown in Figure 1.

### PERG and VEP Data in OAG and Control Eyes

Considering the individual values of PERG and VEP parameters obtained in response to check edges of 60′ and 15′ detected in OAG eyes with respect to the 95% confidence limits derived from control data, delayed 60′ PERG ITs on 32/37 (86.4%) of OAG eyes and reduced 60′ VEP As on 35/37 (94.5%) of OAG eyes were found; all other individual values of 60′ and 15′ PERG and VEP parameters were reduced (60′ and 15′ PERG As, 15′ VEP As) or delayed (60′ and 15′ PERG ITs, 60′ and 15′ VEP ITs and 60′ and 15′ RCTs) on 37/37 (100%) of OAG eyes.

The number of OAG eyes with abnormal values of 60′ and 15′ PERG and VEP responses and relative percentage is reported in Table 1.

When we considered the mean data of 60′ and 15′ PERG responses obtained in the OAG group, IT values were significantly (*p* < 0.01) delayed with respect to those of controls, and A values were significantly (*p* < 0.01) reduced with respect to control ones.

In the OAG group, considering the mean values of 60′ and 15′ VEP responses, ITs were significantly (*p* < 0.01) delayed and As were significantly (*p* < 0.01) reduced when compared with those of controls. In the OAG group, a significant (*p* < 0.01) increase of both 60′ and 15′ RCTs with respect to control ones was detected.

The mean values of 60′ and 15′ PERG and VEP parameters observed in OAG and control groups and the relative statistical analysis between the two groups are reported in Table 1.

**Figure 2:**
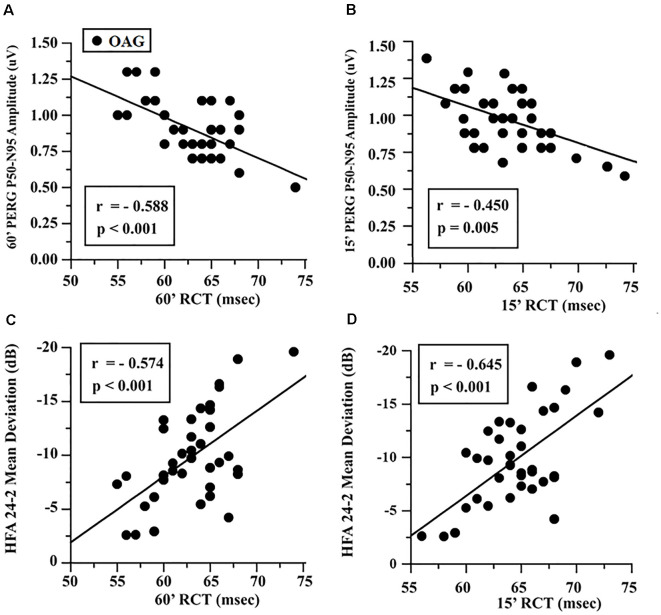
OAG eyes: linear correlation between retinocortical time (RCT) and pattern electroretinogram (PERG) P50-N95 amplitudes **(A,B)** and Humphrey Field Analyzer (HFA) 24-2 mean deviation **(C,D)**. RCT, difference VEP 100 minus PERG P50 implicit times; 60′ and 15′, visual stimuli with checks subtending 60 and 15 min of visual arc, respectively; msec, milliseconds; μV, microvolt.

In the OAG group, the individual values of 60′ and 15′ RCTs were linearly correlated with the corresponding values of PERG A and MD, and it was found that the increase of RCT was significantly (*p* < 0.01) linearly correlated with the reduction of PERG A and of MD values. The values of these linear correlations are reported in Figure 2.

### Retinal Nerve Fiber Layer Thickness Data in OAG and Control Groups and the Correlation With Retinocortical Time in the OAG Group

Considering the individual values of RNFL-T in both temporal and overall evaluation detected in OAG eyes with respect to the 95% confidence limits derived from control data, reduced RNFL-TT or RNFL-OT on 37/37 (100%) of OAG eyes was found. The number of OAG eyes with reduced RNFL-TT or RNFL-OT and the relative percentage are reported in Table 1. On average, in the OAG group, we observed values of both RNFL-OT and RNFL-TT significantly (*p* < 0.01) reduced with respect to control ones.

**Figure 3:**
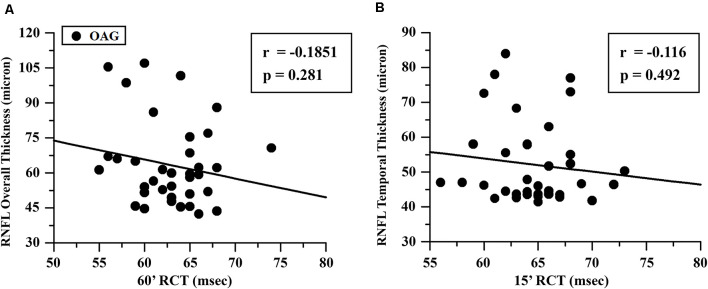
OAG eyes: linear correlation between retinocortical time (RCT) and retinal nerve fiber layer (RNFL) thickness. RCT, difference VEP 100 minus PERG P50 implicit times; 60′ **(A)** and 15′ **(B)**, visual stimuli with checks subtending 60 and 15 min of visual arc, respectively; msec, milliseconds; μ, microns.

The mean values of RNFL-TT or RNFL-OT observed in OAG and control groups and the relative statistical analysis between the two groups are reported in Table 2. In the OAG group, the individual values of 60′ and 15′ RCTs were respectively linearly correlated with the corresponding values of RNFL-OT or RNFL-TT, and it is was found that the increase of 60′ and 15′ RCTs was not significantly (*p* > 0.01) linearly correlated with the corresponding reduced thickness of overall and temporal RNFL. The values of these linear correlations are reported in Figure 3.

**Table 2:** Mean values of retinal nerve fiber layer (RNFL) thickness detected in controls and in patients with open-angle glaucoma (OAG).

	Controls (*N* = 20)		OAG (*N* = 37)		ANOVA: OAG vs. controls		Ab	%
	Mean	1 SD	Mean	1 SD	*F*(1,56) =	*p*=		
RNFL-TT (μ)	85.96	7.62	52.16	11.87	132.09	<0.0001	37	100
RNFL-OT (μ)	110.53	4.56	63.31	17.86	133.89	<0.0001	37	100

*Abbreviations: *N*, number of eyes; SD, standard deviation; ANOVA, one-way analysis of variance; Ab, abnormal with respect to 95% normal confidence limits (mean values −2 SD for RNFL-TT and RNFL-OT); TT, temporal thickness; OT, overall thickness; μ, microns*.

## Discussion

The aims of the present study were to assess the neural conduction along the large and small axons of the postretinal visual pathways and to evaluate whether it was related to RCG function and if it was dependent or not from the RNFL thickness in OAG eyes.

Toward our aim, we assessed in OAG patients simultaneous PERG and VEP recording that allows to derive the RCT, considered an “electrophysiological index” of postretinal neural conduction (Celesia and Kaufmann, 1985; Celesia et al., 1986; Parisi, 1997; Parisi et al., 2001).

The main relevant results of our study were that in OAG the postretinal neural conduction was impaired not exclusively in the component of the small axons, as suggested by the increased 15′ RCT in agreement with our previous findings (Parisi, 1997; Parisi et al., 2001), but the dysfunction involved also the large axons, as detectable by the increased values of 60′ RCT.

In addition, by using visual stimuli of 15′ and 60′ checks (Parisi et al., 2010, 2019a,b; Ziccardi et al., 2013; Barbano et al., 2021), abnormal RCT values were significantly linearly correlated not only with the 15′ PERG As [as previously reported (Parisi, 1997; Parisi et al., 2001)] but also with the 60′ PERG As, thus, meaning that RGC dysfunction influences the postretinal neural conduction along both the small and large axons.

Moreover, both 60′ and 15′ RCT values were significantly linearly correlated with the HFA MD, and this suggests that the severity of glaucomatous visual field damage strictly depends on the abnormal neural conduction from the retina to the visual cortex along both large and small axons.

In addition, since 60′ and 15′ RCTs are derived from 60′ and 15′ VEP IT values, respectively, and based on our previous experience (Barbano et al., 2021), we correlated 60′ RCT values with the corresponding RNFL values obtained from the overall evaluation, representing the average of optic all nerve fibers; by contrast, since the small axons of the papillomacular bundle are located in the temporal sector of RNFL, we correlated 15′ RCT with the corresponding RNFL-TT values.

The interesting and novel finding of this work was that in our OAG patients, the abnormal postretinal neural conduction along the large and small axons (detected by increased 60′ and 15′ RCTs, respectively) was not correlated to the reduced RNFL thickness, as supported by the absence of a significant linear correlation between RNFL-OT and 60′ RCT and between RNFL-TT and 15′ RCT.

The lack of correlation between the parameters describing the postretinal neural conduction and the structure of the axons of the optic nerve in glaucoma requires explanations based on neurophysiological and neuroradiological evidence.

### Neurophysiological Evidence

The neurophysiological mechanisms at the basis of the postretinal neural conduction regard both the formation and stabilization of synapsis between the RGC axons (afferent axons) and the LGN and the integrity of the neural connections between the LGN (efferent axons) and the visual cortex (Hubel and Wiesel, 1965; Parisi et al., 2010).

Indeed, the activity of LGN neural elements is dependent from the neural activation of the afferent axons of RGC, and therefore, a lack or a reduction of bioelectrical activity from the RGC to LGN can produce deficit of LGN neural elements (Parisi et al., 2010). This can be a result of the already reported deprivation of trophic factors (Pease et al., 2000), compromised axonal integrity (Soto et al., 2008), and distal transport (Crish et al., 2010) studied in experimental models of glaucoma.

In our OAG patients, we detected RGC dysfunction, by recordings of abnormal PERG responses, confirming the widely reported evidence in the literature (Parisi et al., 2001, 2006; Ventura et al., 2006; Falsini et al., 2008; Jeon et al., 2019; Jung et al., 2020). The severity of the RGC dysfunction was linearly correlated with the delay of the postretinal neural conduction along both large and small axons. Based on this finding, it is reliable to believe that in our OAG patients, there is a neurophysiopathological condition similar to that previously reported in amblyopia (Parisi et al., 2010) the RCG dysfunction, together with an abnormal structure of their axons (reduced RNFL-T), may induce a reduced bioelectrical activity on the targeted cells of LGN with consequent impairment in the neural afferent input to the LGN of the midbrain (Gupta et al., 2006).

This configures an impairment of the synapsis between RGC and LGN neural elements, thus hypothesizing that glaucoma is not only an axonopathy (Crish et al., 2010; Burgess and Crish, 2018) but also a synaptic pathology at distal sites from the RGC.

On the other hand, in the past, it was assumed that glaucomatous neurodegeneration could depend entirely by local stressors (IOP, genetic profile, vascular impairment, or metabolic dysfunction), and only recently, it has been identified that these etiologic variables contribute to central nervous system (CNS)-modulated apoptosis throughout the visual pathways (Crish et al., 2010; Sponsel et al., 2014). Indeed, damage of both magnocellular and parvocellular layers of the LGN is known in experimental models of glaucoma (Weber et al., 2000; Yücel et al., 2003). The delayed postretinal conduction in OAG can therefore be due also to impairment in the efferent pathways of the LGN, involving the optic radiations and the visual cortex (Crish et al., 2010; Yucel and Gupta, 2015).

Our results are consistent with previous evidence obtained in experimental glaucoma (rat models with acute ocular hypertension) observing an absence of correlations between RGC morphology and visual function evaluated by VEP recordings. In detail, previous works suggested that the degeneration of RGCs can be concomitant to slight VEP amplitude reduction, suggesting possible central compensation (Georgiou et al., 2014) or cortical processing acting to rescue reductions expected from attenuated retinal signals (Tsai et al., 2014).

Therefore, it is reasonable to think that the CNS modulates and orchestrates functional changes that it was suggested to precede apoptosis of RGC (Sponsel et al., 2014, 2017). The neuroplastic remodeling of the ocular dominance columns in the striate cortex, in fact, affirms the control of glaucomatous chronic neurodegeneration by CNS (Reilly et al., 2015), and this may explain also the linear correlation between 60′ and 15′ RCT and HFA MD, leading us to believe that the impaired postretinal neural conduction along both large and small axons may contribute to the glaucomatous visual field defects. On the contrary, the lack of correlation between RCT values and RNFL-T, indicating the absence of a linear correlation between the postretinal neural conduction and the impairment of the RNFL morphology, could be ascribed to several other mechanisms coexisting in glaucomatous neurodegeneration, such as cortico-thalamic feedback (Baroncelli and Lunghi, 2021) and cortical atrophy (Gupta et al., 2006), as well as defective myelination within the optic radiations (Kaushik et al., 2014).

### Neuroradiological Evidence

Neuroimaging methods have been used to explore the possible involvement of different cerebral structures forming the visual pathways in glaucomatous patients. About this topic, several studies have been published; however, we here take into consideration only those more relevant to our findings.

In particular, by using 1.5-T MRI, a reduced size, likely due to a neural degeneration, of the LGN in OAG patients with moderate visual field loss was detected (Gupta et al., 2009), and this study performed in humans was the first, confirming all that was previously observed in experimental animal models (Weber et al., 2000; Yücel et al., 2000). It was interesting to find that a correlation between the LGN atrophy and the clinical stage of the disease was also detected (Dai et al., 2011).

By contrast, by using 3-T MRI, no correlation between the values of LGN neuroradiological parameters and those of visual field or RNFL was found, and this lack of correlation was ascribed to the different neuronal arrangements between the RGC and their fibers and LGN (Furlanetto et al., 2018).

All that was detected by MRI about LGN involvement should be an evidence of the previous suggested impairment of the afferent synapsis from RGC to LGN neural elements with a consequent trans-synaptic degeneration of LGN in glaucomatous optic neuropathy (Lawlor et al., 2018).

In addition to the MRI evidence regarding the LGN, there are several neuroimaging studies showing that in glaucoma there is a morphological involvement of other structures forming postretinal visual pathway including the visual cortex (Chen et al., 2013; Frezzotti et al., 2016; Fukuda et al., 2018).

In particular, by applying a 3-T diffusion tensor MRI, Garaci et al. (2009) noticed an increased diffusion tensor and decreased fractional anisotropy, reflecting axonal disruption of the optic nerves and optic radiations in OAG patients. Moreover, vision-related brain atrophy has been found on both sides of the visual cortex, and it was interesting to detect that other CNS structures (such as the frontoparietal cortex, hippocampus, and cerebellar cortex) may be involved by neurodegenerative process (Nucci et al., 2013; Frezzotti et al., 2014; Giorgio et al., 2018). All this supports the hypothesis that in glaucoma there is a neurodegeneration spreading involving not exclusively the visual pathways but also other brain structures (Hardy and Revesz, 2012).

### Conclusions

In conclusion, in OAG, by appropriate electrophysiological approaches (simultaneous PERG and VEP recordings with the assessment of RCT), it is possible to detect a functional damage of postretinal neural structures.

The observed increased RCTs, recorded by using 60′ and 15′ checks as visual stimuli, indicate that in glaucoma there is a dysfunction involving both postretinal large and small axons. This impairment can be related to the RGC dysfunction (as derived by the linear correlation between the reduction of PERG As and the increase of RCT) and may contribute to the visual field defect (as indicated by the linear correlation between the increase of RCT and the reduction of MD).

By contrast, the lack of correlation between the reduction of RNFL-T and the increase of RCT suggests that the dysfunction involving both postretinal large and small axons is independent from the morphological condition of RGC fibers forming the optic nerve head. All this is consistent with previous findings reporting that in OAG the abnormal neural conduction along the small axons of whole visual pathways (delayed 15′ VEP ITs) was not correlated with the RNFL reduction (Parisi et al., 2001).

The postretinal neural conduction impairment should be explained based on neuropathological processes that may induce the abovementioned (see *PERG and VEP data in OAG and control eyes* and *Retinal nerve fiber layer thickness data in OAG and control groups and the correlation with retinocortical time in the OAG group* sections) functional and structural changes on the postretinal elements, leading to abnormal synaptic connectivity between RGC fibers and LGN and a consequent reduced bioelectrical activity to the visual cortex.

Our findings are in agreement with all recent opinions (see, as review, Kasi et al., 2019) that consider OAG as a neurodegenerative process, in which the involvement is not exclusively located at the level of the neural ocular elements (i.e., RGC) but also an impairment of all visual pathway structures responsible for conveying visual information from the eye to the brain.

Nevertheless, actually, there is a lack of information derived from comprehensive studies in which the brain structural changes, the electrophysiological abnormalities, and the morphological involvement of the retinal structures are evaluated in the same cohort of OAG patients.

This is a challenge for our next future study to provide further information about the mechanisms inducing glaucomatous visual field loss, with consequent innovative therapeutic approaches.

## Data Availability Statement

The raw data supporting the conclusions of this article will be made available by the authors, without undue reservation.

## Ethics Statement

The studies involving human participants were reviewed and approved by Local IRB Comitato Scientifico IRCCS Fondazione Bietti, Rome, Italy. The patients/participants provided their written informed consent to participate in this study.

## Author Contributions

Concept and design, critical revision of the article, and final approval of the article: VP, FO, and GM. Data collection: LZ, LT, GR, LB, and CC. Statistical expertise: VP and FO. Analysis and interpretation, and writing of the article: VP, LZ, GR, FO, and GM. All authors reviewed the manuscript and agreed to be accountable for all aspects of the work. All authors contributed to the article and approved the submitted version.

## Conflict of Interest

The authors declare that the research was conducted in the absence of any commercial or financial relationships that could be construed as a potential conflict of interest.

## Publisher’s Note

All claims expressed in this article are solely those of the authors and do not necessarily represent those of their affiliated organizations, or those of the publisher, the editors and the reviewers. Any product that may be evaluated in this article, or claim that may be made by its manufacturer, is not guaranteed or endorsed by the publisher.
